# The theoretical basis of a nationally implemented type 2 diabetes prevention programme: how is the programme expected to produce changes in behaviour?

**DOI:** 10.1186/s12966-021-01134-7

**Published:** 2021-05-13

**Authors:** Rhiannon E. Hawkes, Lisa M. Miles, David P. French

**Affiliations:** Manchester Centre of Health Psychology, Division of Psychology and Mental Health, University of Manchester, Manchester, UK

**Keywords:** Theory, Intervention design, Logic model, Behaviour change, Type 2 diabetes, Diabetes prevention Programme

## Abstract

**Background:**

It is considered best practice to provide clear theoretical descriptions of how behaviour change interventions should produce changes in behaviour. Commissioners of the National Health Service Diabetes Prevention Programme (NHS-DPP) specified that the four independent provider organisations must explicitly describe the behaviour change theory underpinning their interventions. The nationally implemented programme, launched in 2016, aims to prevent progression to Type 2 diabetes in high-risk adults through changing diet and physical activity behaviours. This study aimed to: (a) develop a logic model describing how the NHS-DPP is expected to work, and (b) document the behaviour change theories underpinning providers’ NHS-DPP interventions.

**Methods:**

A logic model detailing how the programme should work in changing diet and activity behaviours was extracted from information in three specification documents underpinning the NHS-DPP. To establish how each of the four providers expected their interventions to produce behavioural changes, information was extracted from their programme plans, staff training materials, and audio-recorded observations of mandatory staff training courses attended in 2018. All materials were coded using Michie and Prestwich’s Theory Coding Scheme.

**Results:**

The NHS-DPP logic model included information provision to lead to behaviour change intentions, followed by a self-regulatory cycle including action planning and monitoring behaviour. None of the providers described an explicit logic model of how their programme will produce behavioural changes. Two providers stated their programmes were informed by the COM-B (Capability Opportunity Motivation – Behaviour) framework, the other two described targeting factors from multiple theories such as Self-Regulation Theory and Self-Determination Theory. All providers cited examples of proposed links between some theoretical constructs and behaviour change techniques (BCTs), but none linked all BCTs to specified constructs. Some discrepancies were noted between the theory described in providers’ programme plans and theory described in staff training.

**Conclusions:**

A variety of behaviour change theories were used by each provider. This may explain the variation between providers in BCTs selected in intervention design, and the mismatch between theory described in providers’ programme plans and staff training. Without a logic model describing how they expect their interventions to work, justification for intervention contents in providers’ programmes is not clear.

**Supplementary Information:**

The online version contains supplementary material available at 10.1186/s12966-021-01134-7.

Theory has been defined as “a set of concepts, definitions, and propositions that explain or predict events or situations by illustrating the relationships between variables” ([1], p.4). Behaviour change theories identify how and why an intervention should work which subsequently guides intervention development [2]. New guidance from the UK Medical Research Council [3, 4] considers it best practice to apply clear applications of theory in intervention design to understand how interventions should produce changes in behaviour.

There are a number of benefits for the explicit use of theory in intervention design. Firstly, theory will inform interventions by identifying relevant constructs (i.e. key concepts of the theory) that, when targeted, are hypothesised to change behaviour [5]. Secondly, having identified these constructs allows for appropriate intervention techniques, also known as behaviour change techniques (BCTs), to be selected. BCTs have been defined as ‘active ingredients’ of an intervention that can produce behaviour change in an individual, for example self-monitoring, receiving feedback and providing social support [6]. Thirdly, an intervention that has been informed by theory allows developers to conclude why their intervention is effective or ineffective by establishing whether the desired behaviour change was due to changes in intended constructs [7].

Intervention developers often do not clearly specify the theoretical underpinnings of their interventions [8]. Consequently, this limits understanding of what works in changing behaviour and why. To facilitate description on different aspects of theory use, Michie and Prestwich developed the Theory Coding Scheme (TCS) [8]; a 19-item framework designed to assess the extent to which behaviour change interventions are theory-based. The framework provides a fine-grained assessment of the use of theory, including the extent to which all constructs of a theory are targeted by BCTs, and the extent to which all mentioned BCTs are linked to relevant constructs [8]. Despite research efforts to encourage the better reporting of theory, reviews of the behaviour change literature suggest that theory is poorly used in developing and describing such interventions. For example, systematic reviews of interventions for patients with Type 2 diabetes concluded that interventions lacked explicit theoretical frameworks informing their design [9, 10].

A concise way of representing theoretical explanations of behaviour change is via a logic model, which uses simple diagrams to demonstrate the causal pathways between the intervention components and desired outcomes [11]; the theory of the intervention underpins the arrows and shapes in the logic model [12]. Without a logic model, we are unclear why an intervention has been developed in a particular way. Consequently, if the design of an intervention is not clear, staff are unlikely to be trained adequately in the relevant intervention features and, thus, the key techniques hypothesised to result in behaviour change are less likely to be delivered in routine practice. This could lead to a loss in fidelity, i.e. the degree to which an intervention is implemented as intended. A logic model would prevent this drift in fidelity. Therefore, providing a logic model of an intervention is akin to providing a road map of the programme as it highlights how the intervention is expected to work and how the desired outcomes of that intervention are achieved [13].

An intervention informed by a variety of evidence sources is the National Health Service Diabetes Prevention Programme (NHS-DPP). In response to the growing number of people developing Type 2 diabetes worldwide [14], NHS England rolled out the national programme in 2016, aimed at adults in England with elevated blood glucose levels (non-diabetic hyperglycaemia) who are therefore at a higher risk of developing Type 2 diabetes [15]. The programme aims to achieve behaviour change resulting in increased physical activity, improved diet and subsequent weight loss [16] to slow or stop progression to Type 2 diabetes. The NHS-DPP is the largest national diabetes prevention programme globally [17] and has been delivered by four commercial providers for the first three waves of implementation between 2016 and 2019. Of the first 100,000 referrals in the NHS-DPP in 2016–2017, 56% took up a place on the programme and 34% went on to attend the recommended proportion of sessions [18]. See Hawkes et al. [19] for a description of NHS-DPP programme delivery.

Theory use in the NHS-DPP was a key element in the Service Specification that stipulated the minimum requirements that providers had to meet for intervention delivery [16]. Specifically, the Service Specification stated that providers “must be explicit regarding the behaviour change theory and techniques that are being used, and the expected mechanism of action” ([16], p.8), though this document did not specify particular constructs to be targeted in the programme. Providers of the NHS-DPP were required to describe their intervention designs, including theory that provided the basis for their programmes, during service procurement. A previous evaluation assessed the pilot phase and first wave roll-out of the NHS-DPP [20], however, there has yet to be an in-depth analysis of the theory informing providers’ intervention designs.

The authors of the current study have previously identified the BCTs that providers planned to deliver in their programmes [21], but it is not clear how these BCTs were linked to theory. Hawkes et al. [21] established there was variation between the BCTs specified in each providers’ intervention plans. A discrepancy was also identified between the BCTs specified for inclusion in the NHS-DPP full programme specification and the BCTs that providers planned to deliver in their intervention plans [21]. Subsequent research found that providers were not training their staff in all key techniques specified in their intervention plans [22]. Further, there was a notable under-delivery of some BCTs used to self-regulate behaviour (e.g. goal setting, problem solving and self-monitoring of behaviour) in NHS-DPP delivery [23, 24]. There is no explicit logic model of the NHS-DPP to describe expected mechanisms of change, that is, which BCTs the commissioners of the NHS-DPP believe will work in changing physical activity and dietary behaviours, and why.

This study sought to document the use of behaviour change theories underpinning the NHS-DPP. The aims of the current study were to: (a) develop a logic model that underpins the NHS-DPP based on the specification documents detailing how the NHS-DPP is expected to work, and (b) describe the nature and extent of theory use underpinning providers’ NHS-DPP interventions, according to the programme plans and staff training for each provider.

## Methods

### Aim 1: the development of a logic model underpinning the NHS-DPP

#### Document review

Author DPF selected the below specification documents to be reviewed as they provided the basis for NHS-DPP. These documents consisted of:

- Public Health England’s *“Systematic review and meta-analysis assessing the effectiveness of pragmatic lifestyle interventions for the prevention of type 2 diabetes mellitus in routine practice”* [25]. This was chosen as a specification document as the review was originally commissioned to inform the NHS-DPP.
- NHS England’s NHS-DPP Service Specification [16], the commissioning document for the NHS-DPP which draws on recommendations from National Institute of Health and Care Excellence (NICE) PH38 guideline [26]. This document provides a clear indication of what NHS England were looking for in the inclusion of the NHS-DPP.
- NICE PH38 public health guideline [26], “*Type 2 diabetes: prevention in people at high risk*,” was referred to in NHS England’s NHS-DPP Service Specification [16] as it provided additional information regarding behaviour change content to be included in diabetes prevention programmes which was not repeated in the Service Specification [16].

#### Procedures

The three specification documents were reviewed to elicit information on the use of BCTs and expected mechanisms of action to produce a logic model for the NHS-DPP intervention. The following steps were taken:

1. Information on (a) the assumptions underpinning the NHS-DPP (i.e. why specific activities and intervention features are expected to change behaviours), (b) the overall approach that the programme should take (i.e. the general structure of the programme), and (c) BCTs that were specified for inclusion in the programme were extracted into a word document. Information were extracted for each specification document separately (see Additional file 1 for extracted statements from the specification documents).
2. This information was firstly used to create a service logic model, describing what the overall NHS-DPP service should look like, including inputs, outputs, and expected short-term, intermediate and long-term outcomes (see Additional file 2).
3. The NICE PH38 guideline [26] provided the most comprehensive information on the use of BCTs and their expected mechanisms of action, i.e. why specific BCTs were predicted to change physical activity and dietary behaviours in those at risk of Type 2 diabetes. The information extracted from the NICE guideline was firstly compiled into an ‘If-Then’ table, e.g. “if a person-centred and empathy building approach is taken, then individuals will build confidence and self-efficacy over time” (see Additional file 3). The extracted statements from the ‘If-Then’ table provided the basis for indicating which BCTs were expected to change which constructs.

### Aim 2: nature and extent of theory use underpinning the NHS-DPP

#### Design

A document analysis of each of the four providers’ programme plans and staff training materials, and observations of one set of mandatory staff training courses for each of the four providers.

#### Document review

Each provider programme plan consisted of:

- Framework response documents supplied by each provider and submitted during bids for service procurement. These documents described each providers’ proposed service delivery including the programme curriculum, overall structure of their programmes, the planned BCTs and theory that provided the basis for the design of their programmes.

Each providers’ staff training materials consisted of:

- The pre-course reading supplied to trainee facilitators of each of the four providers before they attended mandatory training courses.

Although the providers’ programme manuals described the planned activities and behaviour change content to be delivered to service users on the programme (i.e. guidance detailing activities that facilitators should deliver at each NHS-DPP session), they did not describe any theoretical content *underpinning* each providers’ programmes. Therefore, authors made the decision not to analyse providers’ programme manuals, as they did not detail explicit theoretical principles of that provider’s programme.

#### Participants

The four NHS-DPP providers were private service organisations who each secured contracts to deliver the NHS-DPP in localities across England in 2016–2019. Three of the providers were national organisations who deliver a range of programmes for health, wellbeing and employment (i.e. ICS, Ingeus, and Reed Momenta), and one of the providers was a non-profit organisation (LWTC).

Trainers were employed by each of the four providers or the intervention developers and delivered staff training courses to newly appointed facilitators. Trainee facilitators were required to attend mandatory face-to-face training courses before they were allocated their own diabetes prevention groups to deliver in routine practice.

#### Coding framework

The Theory Coding Scheme (TCS [8];) was used to document theory described in each of the providers’ documents and staff training sessions. The TCS is a 19-item framework which captures whether theory was mentioned, how theory is directly used in the intervention (e.g. links between theoretical constructs and BCTs) and how theory explains intervention effects on outcomes. The authors reported satisfactory inter-rater reliability for each of the items of the TCS [8].

#### Procedures

Authors were in contact with the management staff of each of the four providers to obtain all relevant documentation, including providers’ programme plans and staff training materials. These documents were either emailed or hard copies were sent via post to the research team.

Authors attended one set of mandatory training courses for each of the four providers between February and December 2018. The training sampled was based on the timing of training of each NHS-DPP provider who were recruiting new facilitators and delivering staff training at the time of the evaluation (2018–2019). The four provider training courses were observed in four different geographical areas. The training was between 2 and 5 days for each of the providers, and the training for each provider involved between one and five trainers. Written informed consent was obtained from all participants prior to the training courses commencing. An audio-recorder was placed next to the trainer at the front of the room to capture all training content delivered during the training sessions. This allowed the recording of any information on theoretical principles delivered to trainees during the duration of the training. The present research received ethical approval by the North West – Greater Manchester East NHS Research Ethics Committee on 1st August 2017 (Reference: 17/NW/0426).

A data extraction sheet was developed using Michie and Prestwich’s [8] TCS. Authors removed items 14–19 of the TCS as these items related to post-intervention rather than protocol assessment. Authors also made the decision to add items to the TCS to ensure all relevant theoretical content stated by each provider was captured. The following items were added to the TCS:

- Item 1b: ‘A construct was mentioned’, this was added to capture a construct or predictor that was mentioned but not linked to behaviour.
- Item 7b: ‘All intervention techniques are explicitly linked to an overall theory/model but not a specific construct’.
- Item 8b: ‘At least one, but not all, of the intervention techniques are explicitly linked to an overall theory/model but not a specific construct’.
- Item 9b: ‘Group of techniques are linked to an overall theory/model but not a specific construct’.

#### Analysis

Data were extracted from the relevant documents and audio-recordings using the author-developed data extraction sheet, based on Michie and Prestwich’s [8] TCS. One author (REH) extracted theory detailed in providers’ programme plans onto a separate TCS for each provider. Any underpinning theory detailed in providers’ pre-course reading materials supplied to trainees were extracted onto separate TCS documents. The same author (REH) also extracted data on theory from audio-recorded staff training sessions for each provider using the TCS. Where it was unclear what would be more appropriate to code, the coding was discussed with DPF and consensus agreed. Researcher LMM double-coded providers’ programme plans. Inter-rater reliability (IRR) was calculated using the kappa statistic to determine consistency between coders [27]. Identified coding discrepancies were discussed between REH, LMM and DPF until agreement was met.

The theoretical principles detailed in providers’ programme plans, staff training materials and audio-recorded staff training sessions were summarised for each provider.

## Results

### Logic model underpinning the NHS-DPP

**Fig. 1:**
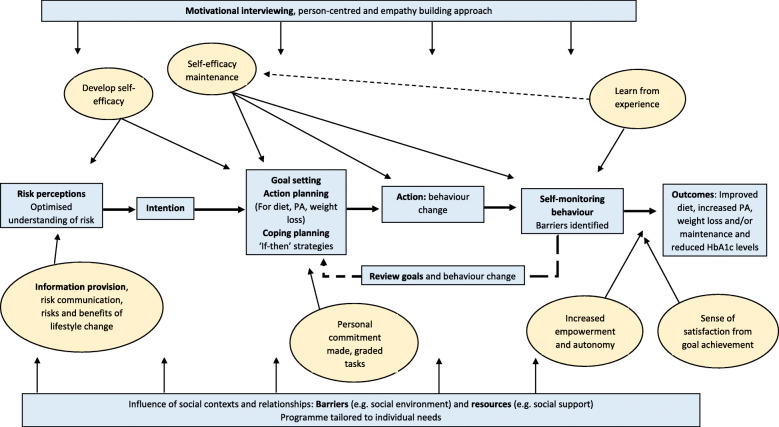
Logic model showing anticipated mechanisms of action of the NHS-DPP

The logic model describing anticipated mechanisms of action of the NHS-DPP is shown in Fig. 1. The figure shows how all constructs relate to the main outcomes of improving dietary behaviours, increasing physical activity and achieving weight loss/maintenance to reduce Type 2 diabetes risk.

Based on the information and BCTs extracted from the NICE PH38 [26] guidance, the first component of the logic model includes initial information provision to service users about their risk of developing Type 2 diabetes, along with the risk and benefits of lifestyle change, which in turn should lead to an intention to change behaviour (component 2). The third component of the model is a self-regulatory cycle of setting achievable short-term and long-term goals for diet, physical activity and weight loss, self-monitoring of behaviours, developing coping strategies, reviewing progress and modifying goals in light of achievement. This in turn should allow the development and maintenance of self-efficacy, learning from experience and resulting in increased autonomy and self-satisfaction of goal achievement. The fourth component is the expected outcomes of increased activity levels, improved diet and corresponding weight loss (see Fig. 1). Throughout the programme, NICE [26] specified a person-centred and empathy building approach should be adopted and an individual’s social context should be taken into account to ensure the programme is tailored to the needs of each service user, which has been reflected in the logic model.

The NICE PH38 guidance [26] stipulated that diabetes prevention programmes should follow a logical progression from information provision, exploration of an individual’s motivation to change, followed by self-regulatory techniques to lead to behaviour change. These processes described by NICE [26] map onto the constructs in the Health Action Process Approach model (HAPA [28];) which recognises these phases of behaviour change, from forming intentions in a motivational phase, to turning them into action and performing the behaviour.

### Theory described in providers’ programme plans

Across the four providers, the mean kappa value for coding using the TCS was 0.92 and the mean kappa values for theories and constructs mentioned were 1.00 and 0.79 respectively. Kappa values demonstrated moderate to almost perfect agreement [27] between coders using the TCS prior to resolving discrepancies (see Additional File 4 displaying all IRR values for the double coding of theory in programme plans).

The extent to which theory was used in each of the four providers’ programme plans is summarised in Table 1. All four providers mentioned theories that their programmes were based on, but none provided an explicit logic model of how their programme will produce changes in behaviour. Providers B and C stated their programmes were informed by the COM-B (Capability Opportunity Motivation – Behaviour) framework [29]. The other two described targeting factors derived from multiple theories, some of which included Leventhal’s Common Sense Model [30], Social Cognitive Theory [31], Self-Determination Theory [32], and Theory of Planned Behaviour [33]. A full list of theories and constructs mentioned by each provider is shown in Table 2.

**Table 1:** Use of theory in provider’s programme plans

	Provider A	Provider B	Provider C	Provider D
Theory mentioned (1a)	✔	✔	✔	✔
Construct mentioned (1b)^*a*^	✔	✔	✔	✔
Target construct mentioned as predictor of behaviour (2)	✔	✔	✔	✔
Intervention based on a single theory (3)		✔	✔	
Theory/predictors used to select recipients for the intervention (4)				
Theory/predictors used to select/develop intervention techniques (5)	✔	✔	✔	✔
Theory/predictors used to tailor intervention techniques to recipients (6)	✔			
All intervention techniques are explicitly linked to at least one theory-relevant construct/predictor (7a)				
All intervention techniques are explicitly linked to an overall *theory/model* but not a specific construct (7b) ^*a*^				
At least one, but not all, of the intervention techniques are explicitly linked to at least one theory-relevant construct/predictor (8a)	✔	✔	✔	✔
At least one, but not all, of the intervention techniques are explicitly linked to an overall *theory/model* but not a specific construct (8b) ^*a*^	✔			✔
Group of techniques are linked to a group of constructs/predictors (9a)				✔
Group of techniques are linked to an overall *theory/model* but not a specific construct (9b) ^*a*^	✔			✔
All theory-relevant constructs/predictors are explicitly linked to at least one intervention technique (10)		✔ ^*b*^	✔	✔ ^*b*^
At least one, but not all, of the theory relevant constructs/predictors are explicitly linked to at least one intervention technique (11)	✔			
Theory-relevant constructs/predictors are measured (12)				
Quality of measures (13)				

Note: Numbers in brackets denote the items of the Theory Coding Scheme (Michie & Prestwich, 2010). Items 14–19 of the Theory Coding Scheme relate to post-intervention not protocol assessment, therefore not included in this analysis

^a^ Additional items which authors added to the Theory Coding Scheme for this analysis

^b^ Provider B linked all theoretical constructs to BCTs for the COM-B model and Provider D links all ‘processes’ (which included a group of constructs) to BCTs for the Process Model of Lifestyle Behaviour Change, however not all mentioned constructs from other theories were linked to BCTs

**Table 2:** Theory and constructs mentioned in each providers’ programme plans and staff training

	Provider A		Provider B		Provider C		Provider D	
	Design	Training	Design	Training	Design	Training	Design	Training
**Model of behaviour mentioned**								
Action planning [34]	✔							
Behaviour Change Wheel [35]			✔					
COM-B Model [29]			✔		✔			
Control Theory [36]							✔	
Dual Processing Theory [37]	✔	✔						
Health Action Model [38]				✔				
Health Belief Model [39]				✔				
Leventhal’s Common Sense Model [30]	✔	✔						
“Positive Model of Good Health” [40]			✔					
Self-Regulation Theory [41]	✔	✔					✔	
Self-Determination Theory [32]							✔	
Social Cognitive Theory [31]	✔						✔	
Social Learning Theory [42]		✔						
Stages of Change Model [43]				✔				
Theory of Planned Behaviour [33]				✔			✔	
The Process Model of Lifestyle Behaviour Change [44]							✔	✔
**Constructs mentioned**								
Attitudes			✔					
Behavioural regulation			✔		✔		✔	
Capability			✔		✔			
Cognitive illness representations	✔	✔						
Consequences	✔	✔						
Coping Styles			✔					
Costs/benefits							✔	
Descriptive norms			✔		✔			
Goals	✔		✔		✔			
Intentions			✔					
Intrinsic motivation, social support, competence							✔	
Health beliefs	✔		✔	✔			✔	
Knowledge acquisition			✔					
Motivation			✔	✔	✔			
Motivation, action and maintenance processes							✔	
Pre-contemplation, contemplation, preparation, action, maintenance				✔				
Risk perceptions							✔	
Opportunity			✔		✔			
Problem solving abilities			✔					
Self-efficacy	✔	✔	✔	✔	✔		✔	
Social environment	✔							
Social influences			✔					
Vicarious experience	✔	✔						

Note: Staff training includes the pre-course reading and face-to-face core training sessions

Although all four providers described a number of BCTs relating to theoretical constructs (item 8a of TCS; see Table 1), no provider gave explicit detail about how these connections were determined and none explicitly linked all specified techniques to theoretical constructs (item 7a in the TCS). Providers B and C linked all the constructs of the COM-B model (capability, opportunity, motivation [29];) to intervention techniques (item 10 of the TCS; see Table 1). One target construct, self-efficacy, was mentioned by all providers in their programme plans, and three providers explicitly linked self-efficacy to intervention techniques.

### Theory described in providers’ staff training

Providers varied in the extent to which they trained staff in the theoretical principles underpinning their programmes. Pre-course reading supplied by each provider also varied, including journal articles, pre-course manuals and reading to supplement the programme manuals. Only providers A and B supplied trainees with documents detailing some theoretical underpinning (see Table 2 for all theory described in providers’ staff training).

Each provider had a different number of mandatory training days that staff were required to attend, ranging from 2 to 5 days. The final sample of NHS-DPP staff training consisted of 13 mandatory training days across the four providers. All attending trainers (*n* = 10) and trainees (*n* = 78) consented to the researchers attending, observing and audio-recording the NHS-DPP staff training courses. Trainers had backgrounds including public health, nutrition, personal training and diabetes care.

In the face-to-face training, Provider A included discussions about the underlying theory of some activities. For example, the elicitation of service user health beliefs was informed by Leventhal’s Common Sense Model [30] and Social Learning Theory [42] underpinned activities that aimed to build service user confidence in managing their health. Providers C and D did not describe any theory that their programmes were based on during in the face-to-face training, though Provider D supplied a diagram of The Process Model of Lifestyle Behaviour Change [44] in accompanying materials, but no further explanation was provided (see Table 2 for theory described in staff training).

### Theoretical principles described in programme plans vs. staff training

There were some discrepancies between the theory described in providers’ programme plans and the theory described in the staff training (see Table 2). For example, Provider B stated in their plans that their programme was based on the COM-B model [29], however they supplied a training document about the Transtheoretical Model [43] underpinning goal setting, even though this model was not described in their programme plans. The only provider who detailed consistent theory in both their programme plans and staff training was Provider A (see Table 2).

## Discussion

Authors of this paper constructed a logic model to describe why the NHS-DPP should work in changing dietary and physical activity behaviours, using inference from the three specification documents underpinning the NHS-DPP, as commissioners had not previously produced a logic model. As a possible result of a logic model not being specified, a variety of different behaviour change theories are used by each provider, and none provided an explicit logic model of how they expect their intervention to work. Without a clear logic model, justification for the intervention contents of each providers’ NHS-DPP programme is not clear. This may explain the variation in planned BCTs between providers, which do not map onto the BCTs specified for inclusion by NHS England [21].

Although providers mentioned theory in their intervention plans, they did not explicitly demonstrate all links between planned BCTs and the proposed targeted constructs. The absence of one-to-one mapping between each BCT and theoretical construct is a sub-optimal way to report theory [8], as it does not describe how providers will change intended constructs. Further, discrepancies were identified between theory that providers described in their programme plans and theory that they trained their staff in. Both of the above could be a consequence of not having a logic model from the outset to describe how providers expect their interventions to work.

### Strengths and limitations

There are a number of strengths of the current study. We obtained all the documentation that providers were required to supply to NHS England during the process of bidding for service procurement, and all relevant pre-course reading materials supplied to trainees were also obtained from each provider. Authors used more recent documentation compared to a previous evaluation of the pilot phase and first wave roll-out of the NHS-DPP [20], and the use of a standardised tool to code for theory (TCS [8];) allowed for a more fine-grained assessment of theory compared to the previous evaluation.

However, this study is not without its limitations. Authors had to base the current analysis on explicit theoretical links stated by providers. For example, in providers’ programme plans, BCTs were sometimes mentioned in close proximity to constructs or theories but were not explicitly linked. Authors did add some items to the TCS framework [8] to ensure that all theoretical content suggested by providers was captured. Secondly, although there were no explicit logic models supplied by providers in their framework response bids, we cannot know whether providers did develop their own logic models which we are unaware of, or whether providers only stated arbitrary reasons for the inclusion of particular techniques in their intervention.

Despite this, none of the providers supplied any further documentation on theoretical content. Authors were justified in only assessing providers’ framework response documents for assessing the design of their interventions as these were submitted in providers’ bids for service procurement and NHS England explicitly required providers to specify theory, BCTs and mechanisms of action in these documents [16]. Thus, all relevant information regarding theory should have been available in the framework response bids. This highlights the complexities of conducting a programme evaluation as external evaluators; as authors were not involved in the development of the interventions, we did not know the exact processes and underlying logic of providers’ intervention designs.

Finally, authors only observed one set of mandatory face-to-face staff training courses for each provider. Thus, we cannot know whether the same results would have been obtained from another sample of training courses. However, the same pre-course reading materials would have been supplied to all trainee facilitators of each provider. Given that two providers lacked explicit theoretical content in these materials, it is possible that the training of underpinning theory may have not been present in the mandatory training courses either.

### Relation to existing research

Providers’ NHS-DPP interventions appear to describe ‘evidence-inspired’ approaches rather than theory-based [45]. That is, their programme plans outlined some description of theory, but providers were not explicit in how the proposed theories were used, thus highlighting a lack of justification for BCT selection. Consequently, we cannot ascertain which intervention techniques work and how exactly they work in reducing the incidence of Type 2 diabetes in those at risk [45]. This highlights the limitation of not developing an explicit logic model. Our findings are in line with previous reviews of behaviour change interventions which also reported a lack of theory reporting in intervention designs [9, 10, 46–48].

Given the lack of justification for some of the providers’ theoretical underpinnings, it appears that providers are not systematically selecting theories to underpin their interventions. Consequently, some of these theories have less of a focus on self-regulation techniques such as coping planning and self-monitoring, which the NICE guidance informing the NHS-DPP emphasises [26]. For example, the Theory of Planned Behaviour [33] and the Health Belief Model [39] fail to acknowledge the post-intentional and self-regulation components during the volitional stages of making behavioural changes. However, this is a strong component described in the NICE guidance [26] and therefore included in the logic model developed by the current authors. The only model cited by one of the providers which addresses this volitional stage of self-regulation is the Process of Lifestyle Behaviour Change [44] which is an adaptation of the HAPA model [28].

Boulton et al. [49] provides an illustration of how theory can be mapped at the intervention design stage. The authors developed an intervention to increase physical activity and improve balance and strength in adults aged 60–70 years. The intervention was explicitly based on the HAPA model [28]; authors mapped links between the HAPA constructs and their intervention, all elements of the intervention were mapped onto BCTs from the BCT Taxonomy v1 [6]. This detailed mapping enabled the authors to develop, test and improve their physical activity intervention, describing the optimal way of developing theory-based interventions [8]. There is at least some evidence that the importance of theory for behaviour change in the NHS-DPP is acknowledged; NHS England did at least specify that behaviour change theory must be explicitly described, and the four providers did respond to this, albeit variably. However, it is clear that more can be done to optimise the reporting of underpinning theory in the NHS-DPP.

### Implications for practice and research

The fact that providers are using a variety of behaviour change theories in different ways with variation in planned BCTs does not facilitate a clear roadmap on what their interventions are trying to do. Consequently, staff are less likely to be trained in these key intervention techniques, which would decrease the likelihood of these techniques being delivered in routine practice. A logic model would prevent this drift in fidelity. Further, as Kirk et al. [50] explain, an intervention cannot be adapted for particular contexts unless the underlying theory and causal mechanisms are understood. This would ensure that different forms of the intervention (i.e. the adaptations) are performing the same function (i.e. the causal mechanisms for behaviour change). In other words, developing a logic model to understand the key mechanisms in changing behaviour (the function) will allow providers to adapt their intervention to local context without sacrificing fidelity [12].

It seems that providers did not receive much initial guidance from NHS England with regards to the constructs to be targeted in the NHS-DPP and specific components for the logic model, although BCTs were specified. To address this, future implementations of large-scale programmes could benefit from providing an initial logic model from the outset, in accordance with the evidence base, to guide providers to adapt the logic model for their own programmes. Alternatively, the construction and justification of a logic model by each provider could be part of the commissioning process.

Future diabetes prevention programmes could be tested and evaluated more effectively if intervention developers incorporated a logic model at the intervention design stage to establish what works in an intervention and why, which would allow the refinement of the intervention to produce better health outcomes. An alternative would be to ensure a robust quality assurance process, starting from the tender process, to help detect gaps in the plans submitted by providers during tender. Further, if intervention developers were clear on the techniques they are using to target specific constructs in the intervention, an analysis of receipt of these techniques would be able to determine whether the intervention is changing the behaviours it intended to change. That is, whether recipients of the intervention understood the intervention content to make the desired behaviour changes.

Future research could conduct interviews with stakeholders who were involved in the design and development of each providers’ NHS-DPP interventions to gain a more in-depth understanding of the theory informing providers’ programmes, justification for the included techniques, and the journey from design through to implementation. Authors of the current paper are pursuing this method for the evaluation of the digital NHS-DPP.

## Conclusions

The NHS-DPP has been designed to target multiple predictors of behaviour and a variety of behaviour change theories are used in different ways by the providers. This has resulted in variation of planned BCTs across providers, which do not map onto the BCTs specified for inclusion by NHS England [21]. Without a logic model describing clear one-to-one mapping of all BCTs to theoretical constructs (and vice versa), we cannot be sure of how providers’ interventions expect to produce changes in dietary and physical activity behaviours, that is, their expected mechanisms of change. More consistent use of theory in this national programme would produce greater clarity in intervention contents and would prevent a drift in fidelity in intervention implementation from design through to delivery.

## Supplementary Information

**Additional file 1.** Extracted assumptions for logic model.

**Additional file 2.** NHS-DPP service logic model.

**Additional file 3.** ‘If-Then’ table to inform the BCT logic model of the NHS-DPP.

**Additional file 4.** Kappa values for double coding using TCS framework.

## Data Availability

The data extracted from the specification documents to produce the logic model described in this manuscript are included in this published article and/or supplementary information files. The programme plan documents from independent providers and audio-recordings analysed in the current study are not publicly available due to confidentiality agreements with the provider organisations, as some information is commercially sensitive. Some datasets are available from the corresponding author on reasonable request, although authors will require the explicit permission of the relevant provider organisations.

## Abbreviations

HAPA: Health Action Process Approach model
NICE: National Institute of Health and Care Excellence
NHS-DPP: National Health Service Diabetes Prevention Programme
TCS: Theory Coding Scheme
