# Supplementary material for: The theoretical basis of a nationally implemented type 2 diabetes prevention programme: how is the programme expected to produce changes in behaviour?
Source: Int J Behav Nutr Phys Act. 2021 May 13;18:64. doi: 10.1186/s12966-021-01134-7 (PMC8117267; doi:10.1186/s12966-021-01134-7)
Supplement: Supplementary file 1 — Additional file 1. Extracted assumptions for logic model. [file 12966_2021_1134_MOESM1_ESM.docx]

**change advice includes:**

- Helping people to understand the short, medium and longer-term consequences of health related behaviour
- Helping people to feel positive about the benefits of changing their behaviour
- Building the person's confidence in their ability to make and sustain changes
- Recognising how social contexts and relationships may affect a person's behaviour
- Helping plan changes in terms of easy steps over time
- Identifying and planning for situations that might undermine the changes people are trying to make (including planning explicit 'if–then' coping strategies to prevent relapse)
- Encouraging people to make a personal commitment to adopt health-enhancing behaviours by setting (and recording) achievable goals in particular contexts, over a specified time
- Helping people to use self-regulation techniques (such as self-monitoring, progress review, relapse management and goal revision) to encourage learning from experience
- Encouraging people to engage the support of others to help them to achieve their behaviour change goals

**Extracted from NICE PH38 (2012) – evidence that this guideline is based on**

- A focus on behavioural intention and evidence of **strategies that were common to more than one theoretical model.** These include: providing information and tailoring programmes to individual needs; using multiple sessions to reinforce information; delivery to small groups or individuals; delivering written information as well as verbal advice; **encouraging self-monitoring; and logging of physical activity, diet and weight change**
- For **dietary behaviour change, taking small steps and providing both observational and vicarious leaning opportunities as well as encouraging the identification of barriers and problem-solving** were reported as strategies used in prevention programmes that had achieved reduction in diabetes incidence. For **physical activity, a prescriptive approach that gradually increased the frequency and volume of activity over time as well as providing observational and vicarious learning opportunities and encouraging self-monitoring** were suggested
- **Regular reinforcement of set goals** was reported as an important strategy in the early stages of an intervention
- **Self-monitoring through the use of regular weighing, and recorded measurement of dietary input and physical activity increased self-efficacy and empowerment**. Family was a key social support in prevention efforts
- Feeling better or fitter following the accomplishment of change helped sustain physical activity behaviour changes; a sense of **satisfaction expressed by participants that had achieved their goals**
- **Motivational interviewing**
- Attention to the optimal timing of information-giving allowed gradual absorption of change and therefore was a facilitator in allowing adjustment to changes
- **Increased autonomy and control over behaviour** in those able to manage their weight; **motivate themselves and plan their own lifestyle without the aid of a clinician or adviser.** **Self-efficacy is an important factor in changing behaviour**. **Self-monitoring was a way of keeping to plans and allowing a balance between optimal and realistic goals**

**Extracted from NHS Service Specification (2016) – overall approach and BCTs to be used**

- The content of the sessions should aim to **empower people at risk of diabetes to take a leading role in instituting and maintaining long-term behaviour changes**
- The Provider must ensure that all sessions and communications incorporate clear, targeted, and **high quality communication of risk**, which optimise understanding of the risk of developing Type 2 diabetes and how this can be prevented
- **The Provider must ensure that achievable goals for weight loss** (for those who are overweight or obese) are agreed for different stages of the Service for example, within the first few weeks, at three months and at completion of the Service
- **Service users should set short, medium and long-term goals**
- **The Provider will take a graded and structured approach to setting, monitoring and reviewing goals to ensure that those who have a very low baseline level of physical activity are supported to attain the CMO recommendations within a personalized timeframe**
- **Physical activity should be self-reported and measured, and self-monitoring is especially important for the digital programme**

**Extracted from Ashra et al. (2015) – overall approach**

- Desired outcome: diabetes prevention in the UK – reducing weight and glucose levels in those who are high risk
- Majority of cost to NHS (£10 billion) is due to preventable complications associated with diabetes
- Developing diabetes is highly reversible via weight loss and increased PA levels
- Synthesised the results from 36 studies assessing diabetes prevention programmes in a real-world setting
- Evidence for inclusion in programme theory:
- Utilising a combined diet and PA intervention was associated with greater weight loss than using a PA only intervention
- To maximise reduction in T2DM incidence rate and intervention sessions should be spread across 9-18 months, although weight loss was maximised when the session spread is different to 9-18 months
- The optimum session length to aid greater weight loss was between 1 to 2 hours long
- The total number of sessions over the first 18 months should match or exceed 13 in order to maximise weight loss
- Sixteen or more hours of contact should be provided during the first 18 months to maximise weight loss and fasting glucose reductions
- **Incorporating three or more behaviour change techniques into the prevention programme resulted in smaller reductions in 2-hour glucose than using fewer than three techniques**
- **Use of self-regulatory – monitoring own progress – techniques was associated with a smaller reduction in 2-hour glucose than not using such methods**
- For group delivered interventions the ideal group size was between 10 to 15 individuals in order to maximise weight loss
- Using an evidence base different to the major prevention programmes (DPS or DPP) resulted in smaller weight loss, when compared to using the DPP as the sole evidence base
