# Supplementary material for: The theoretical basis of a nationally implemented type 2 diabetes prevention programme: how is the programme expected to produce changes in behaviour?
Source: Int J Behav Nutr Phys Act. 2021 May 13;18:64. doi: 10.1186/s12966-021-01134-7 (PMC8117267; doi:10.1186/s12966-021-01134-7)
Supplement: Supplementary file 2 — Additional file 2. NHS-DPP service logic model. [file 12966_2021_1134_MOESM2_ESM.docx]

**Additional File 2: NHS-DPP Service Logic Model**

*Note: The boxes in orange were based on data extracted from the Ashra et al. (2015) systematic review and meta-analysis and the NHS-DPP Service Specification (2016). The boxes in green were based on data extracted on behaviour change content from the NICE PH38 (2012) guideline.*

*See below.*

References:

Ashra NB, Spong R, Carter P, Davies MJ, Dunkley A, Gillies C. A systematic review and meta-analysis assessing the effectiveness of pragmatic lifestyle interventions for the prevention of type 2 diabetes mellitus in routine practice. London: Public Health England. 2015. <https://assets.publishing.service.gov.uk/government/uploads/system/uploads/attachment_data/file/456147/PHE_Evidence_Review_of_diabetes_prevention_programmes-_FINAL.pdf>

NHS England. Service Specification No. 1: Provision of behavioural interventions for people with non-diabetic hyperglycaemia. [Version 01]. 2016. <https://www.england.nhs.uk/wp-content/uploads/2016/08/dpp-service-spec-aug16.pdf>

National Institute for Health and Care Excellence (NICE). PH38 Type 2 diabetes: Prevention in people at high risk. London: National Institute for Health and Care Excellence (Updated September 2017). 2012. <https://www.nice.org.uk/guidance/ph38/resources/type-2-diabetes-prevention-in-people-at-high-risk-pdf-1996304192197>

**LONG-TERM
OUTCOMES**

**INTERMEDIATE OUTCOMES**

**INPUTS**

**OUTPUTS/ACTIVITIES**

**SHORT-TERM OUTCOMES**

**National:**

- National independent commercial providers
- National systems for recording attendance, weight, HbA1c, demographics data (minimum dataset)

**Session activities:**

- Diet and PA intervention to target weight loss
- Appropriate, targeted materials – tailored to local population needs

**Resources:**

- NHS England funding
- Competent and qualified educators/staff

**Local:**

- Local population health needs
- Local health economies and community sector organisations

**Referral:**

- Individuals with HbA1c ≥42mmol/mol, over the age of 18 years, not pregnant

**Referral:**

- Individuals with HbA1c ≥ 42mmol/mol, over the age of 18 years, not pregnant

**Face-to-face intervention:**

- Minimum of 13 sessions, lasting 1-2 hours, tapered across 9-18 months
- Minimum 16 hours contact
- Group sizes of 10-15 people
- One-to-one support included

**OR**

**Digital intervention (new Service Specification framework, 2019):**

- When face-to-face service is not accepted
- Intervention as above for 9 months

**Individual:**

- Improved knowledge and attitudes towards diabetes prevention
- Improved knowledge about UK dietary and physical activity recommendations
- Improved social support

**Individual:**

- Continued programme attendance
- Risk-reducing lifestyle behaviour changes
- Individual empowered to take a leading role in maintaining behavioural changes
- Improved ownership of lifestyle changes, e.g. monitoring of own health and working towards long-term goals
- Improved confidence and self-efficacy to make desired changes
- Increased autonomy and control over behaviour
- Individual learning from experience
- Sense of satisfaction from goal achievement

**Individual:**

- HbA1c <42mmol/mol
- Weight loss
- Maintenance of behaviour changes
- Achievement of UK dietary recommendations
- Achievement of the England Chief Medical Officer’s physical activity recommendations
- Confidence to maintain lifestyle changes
- Improved quality of life
- Improved overall health

**National:**

- Reduced incidence of T2DM
- Reduced mortality
- Reduced pressure on the NHS
- Reduced NHS spending on complications associated with T2DM

**Behaviour change techniques:**

- Self-regulatory BCTs, tailored to individuals (e.g. self-monitoring, goal setting, receiving feedback, reviewing progress)

**Individual:**

- Improved ownership of health
- Individual empowered to take a leading role in initiating behavioural changes
- Identification of individual health goals and plan in place to achieve those goals; individuals make a personal commitment

**Underpinning evidence:**

- Systematic review and meta-analyses on effectiveness of DPPs (Ashra et al., 2015)
- NICE guidance, including PH38
- NHS Service Specification – minimum guidelines of what should be present in programme
