# Supplementary material for: The theoretical basis of a nationally implemented type 2 diabetes prevention programme: how is the programme expected to produce changes in behaviour?
Source: Int J Behav Nutr Phys Act. 2021 May 13;18:64. doi: 10.1186/s12966-021-01134-7 (PMC8117267; doi:10.1186/s12966-021-01134-7)
Supplement: Supplementary file 3 — Additional file 3. ‘If-Then’ table to inform the BCT logic model of the NHS-DPP. [file 12966_2021_1134_MOESM3_ESM.docx]

**Additional File 3: ‘If-Then’ Table to Inform the BCT Logic Model of the NHS-DPP**

*Note: Extracted data in the ‘If’ and ‘Then’ rows of the table was taken directly from the NICE PH38 (2012) guideline which gave more detailed information about the behaviour change content that should be included in the intervention. Extracted data in the ‘So That’ and ‘Therefore’ rows of the table were taken from the Ashra et al. (2015) systematic review and meta-analysis and the NHS-DPP Service Specification (2016).*

References:

Ashra NB, Spong R, Carter P, Davies MJ, Dunkley A, Gillies C. A systematic review and meta-analysis assessing the effectiveness of pragmatic lifestyle interventions for the prevention of type 2 diabetes mellitus in routine practice. London: Public Health England. 2015. <https://assets.publishing.service.gov.uk/government/uploads/system/uploads/attachment_data/file/456147/PHE_Evidence_Review_of_diabetes_prevention_programmes-_FINAL.pdf>

NHS England. Service Specification No. 1: Provision of behavioural interventions for people with non-diabetic hyperglycaemia. [Version 01]. 2016. <https://www.england.nhs.uk/wp-content/uploads/2016/08/dpp-service-spec-aug16.pdf>

National Institute for Health and Care Excellence (NICE). PH38 Type 2 diabetes: Prevention in people at high risk. London: National Institute for Health and Care Excellence (Updated September 2017). 2012. <https://www.nice.org.uk/guidance/ph38/resources/type-2-diabetes-prevention-in-people-at-high-risk-pdf-1996304192197>

**Table to Inform Logic Model for Inclusion of BCTs in DPP**

| **IF:** |
| --- |
| - Information and practical tools on nutrition, physical activity and weight management are provided at optimal timing, - High-quality communication of risks is provided, and - There is a discussion about the risks and benefits of lifestyle change |
|  |
| - A person-centred and empathy building approach is taken, including motivational interviewing |
|  |
| - Short, medium and long-term goal setting and action planning (including gradual steps in behaviour change) are agreed over different stages of the service, and - A graded and structural approach to setting, monitoring and reviewing goals is taken |
|  |
| - Regular reinforcement of set goals is provided, and self-monitoring of behaviour and reviewing progress to achieve goals is encouraged, - Coping plans and relapse prevention (including if-then strategies) are put in place, and - Individuals are encouraged to identify and solve problems, overcome barriers and revise goals and action plans accordingly |
|  |
| - Social support is provided from family, friends and/or carers, including practical and emotional support |
| **THEN:** |
| - Individuals will understand the short, medium and long-term consequences of behaviour, with a gradual absorption of change and adjustment, - Individuals will have an optimised understanding of the risk of developing T2DM and how this can be prevented, and - Individuals will feel more positive making lifestyle behaviour changes |
|  |
| - Individuals will build confidence and self-efficacy over time |
|  |
| - Individuals will be encouraged to make a personal commitment to adopt health-enhancing behaviours, and - Individuals will meet dietary and PA recommendations within a personalised timeframe |
|  |
| - Individuals will review their lifestyle behaviours and learn from their experiences, which will increase self-efficacy and empowerment and allow balance between optimal and realistic goals, and - Helping to prevent a relapse |
|  |
| - Individuals will be aided in achieving their behavioural goals over time |
| **SO THAT:** |
| - Individuals achieve a HbA1c of <42mmol/mol, - Individuals achieve weight loss and/or maintenance, - Individuals achieve UK dietary recommendations and Chief Medical Officer’s physical activity recommendations, - Individuals have an improved quality of life and overall health, and - Individuals have confidence to maintain and continue lifestyle behavioural changes |
| **THEREFORE:** |
| - There is a reduced incidence of T2DM, - There are reduced mortality rates, and - There is a reduced pressure on the NHS, including complications associated with T2DM |
