# Supplementary material for: The theoretical basis of a nationally implemented type 2 diabetes prevention programme: how is the programme expected to produce changes in behaviour?
Source: Int J Behav Nutr Phys Act. 2021 May 13;18:64. doi: 10.1186/s12966-021-01134-7 (PMC8117267; doi:10.1186/s12966-021-01134-7)
Supplement: Supplementary file 4 — Additional file 4. Kappa values for double coding using TCS framework. [file 12966_2021_1134_MOESM4_ESM.docx]

**Additional File 4: Kappa Values**

**Table S1. Kappa Values for Theory Coding of Providers’ Programme Plans**

| **Source Document** | **Kappa Value** |
| --- | --- |
| **Provider A Framework Response** | |
| Theory Coding Scheme | 1.00 |
| Theories mentioned | 1.00 |
| Constructs mentioned | 0.64 |
| **Provider B Framework Response** | |
| Theory Coding Scheme | 0.92 |
| Theories mentioned | 1.00 |
| Constructs mentioned | 0.94 |
| **Provider C Framework Response** | |
| Theory Coding Scheme | 0.96 |
| Theories mentioned | 1.00 |
| Constructs mentioned | 0.82 |
| **Provider D Framework Response** | |
| Theory Coding Scheme | 0.80 |
| Theories mentioned | 1.00 |
| Constructs mentioned | 0.75 |
